# A Perspective: Active Role of Lipids in Neurotransmitter Dynamics

**DOI:** 10.1007/s12035-019-01775-7

**Published:** 2019-10-08

**Authors:** Pekka A. Postila, Tomasz Róg

**Affiliations:** 1 Department of Biological and Environmental Science, University of Jyvaskyla, P.O. Box 35, FI-40014 Jyväskylä, Finland; 2 Structural Bioinformatics Laboratory, Biochemistry, Faculty of Science and Engineering, Åbo Akademi University, FI-20500 Turku, Finland; 3 Department of Physics, University of Helsinki, P.O. Box 64, FI-00014 Helsinki, Finland

**Keywords:** Synaptic neurotransmission, Neurotransmitter, Synaptic receptor, Membrane-based sorting, Molecular dynamics (MD), Membrane lipid composition (MLC)

## Abstract

Synaptic neurotransmission is generally considered as a function of membrane-embedded receptors and ion channels in response to the neurotransmitter (NT) release and binding. This perspective aims to widen the protein-centric view by including another vital component—the synaptic membrane—in the discussion. A vast set of atomistic molecular dynamics simulations and biophysical experiments indicate that NTs are divided into membrane-binding and membrane-nonbinding categories. The binary choice takes place at the water-membrane interface and follows closely the positioning of the receptors’ binding sites in relation to the membrane. Accordingly, when a lipophilic NT is on route to a membrane-buried binding site, it adheres on the membrane and, then, travels along its plane towards the receptor. In contrast, lipophobic NTs, which are destined to bind into receptors with extracellular binding sites, prefer the water phase. This membrane-based sorting splits the neurotransmission into membrane-independent and membrane-dependent mechanisms and should make the NT binding into the receptors more efficient than random diffusion would allow. The potential implications and notable exceptions to the mechanisms are discussed here. Importantly, maintaining specific membrane lipid compositions (MLCs) at the synapses, especially regarding anionic lipids, affect the level of NT-membrane association. These effects provide a plausible link between the MLC imbalances and neurological diseases such as depression or Parkinson’s disease. Moreover, the membrane plays a vital role in other phases of the NT life cycle, including storage and release from the synaptic vesicles, transport from the synaptic cleft, as well as their synthesis and degradation.

## Synaptic Membrane Plays a Role in Neurotransmission?

From the traditional viewpoint, the synaptic membrane and its lipids are acknowledged to play a crucial and yet somewhat passive role in the synaptic neurotransmission.

The two adjacent neurons in the synapse can communicate with each other via chemical messengers or neurotransmitters (NTs) that burst out of the presynaptic membrane upon the arrival of the action potential, diffuse across the synaptic cleft and eventually bind into the receptors embedded at the postsynaptic membrane [1, 2]. Accordingly, the opposing membranes not only form barriers between the nerve cells but also house very specific membrane-embedded protein machineries such as receptors, voltage-gated ion channels, transporters, and intracellular proteins (so-called postsynaptic density) that modulate and relay the message across the cleft [3]. It is estimated that about 6600 unique proteins are present in the synapse [4], ensuring tight regulation of the signal transduction [5–7].

As a result, the role of lipids in the synaptic neurotransmission is predominately discussed in the context of their interactions and cooperation with these membrane proteins. Four prominent examples of active cooperation between lipids and proteins in the synapses are given below to demonstrate the wealth of data on the subject.

Firstly, lipids are known to be involved in the regulation of synapse development and plasticity. Tropomyosin receptor kinase B (TrkB) [8], which is a crucial protein in the synapse development, is regulated by cholesterol (CHOL) levels, i.e., losing its activity in membranes with low or high CHOL concentration [9]. Since CHOL levels increase in neurons during development reaching five times higher concentration in the adult brain compared with the early developmental stage [10], the highest level of TrkB activity is limited to a narrow time window of the brain development [11].

Secondly, lipids are heavily involved in presynaptic vesicle release [12–14]—at least 36 protein species are under lipid regulation in this process [12]. Phosphatidylinositols (PIPs), including PI(4,5)P2, PI(3,4,5)P3, PI4P, and other charged lipid species, are responsible for the recruitment of numerous proteins at the presynaptic membrane [12, 13, 15, 16]. The paramount changes in the bilayer curvature during the vesicle fusion/fission are achieved via CHOL translocations between the leaflets or changes in the size ratio of the headgroups to hydrocarbon chains (thus lipid shape) [13, 17]. Various phospholipases actively modulate lipid shape by synthetizing phosphatidic acid (PA) and di-acyl-glycerol (DAG), because lipid species with small headgroups promote negative membrane curvature. Phospholipases also produce lysophosphatidylcholine that has only a single hydrocarbon tail thats promotes positive membrane curvature. The curved regions of the lipid bilayer are specifically recognized by sensory proteins that contain the banana-shaped BAR (Bin Amphiphysin Rvs) domains [18].

Thirdly, neurotransmitter receptors are also regulated by lipids, mostly by direct interactions [19–23], e.g., cholesterol has been shown to function as a direct allosteric regulator of G protein-coupled receptors (GPCRs) [24, 25]. Polyunsaturated fatty acids (PUFAs) are essential in brain functions [26], including the regulation of GPCR oligomerization [27].

Fourthly, lipids function as protein structural elements, and, for this reason, they are frequently found in the X-ray crystal structures of membrane proteins (see Enkavi et al. 2019 [28]). For example, CHOL is clearly visible in the 3D structures of serotonin [29], cannabinoid [30], μ-opioid [31], κ-opioid [32], muscarinic acetylcholine [33], and adenosine [34] receptors, as well as serotonin [35] and dopamine [36] transporters. These co-crystallized or conserved lipids are likely only the tip of the iceberg because the harsh conditions of the protein preparation during crystal preparation get rid of most lipids. Finally, lipidation or the process of covalently attaching lipid groups into membrane proteins, namely palmitoylation, and prenylation, affects numerous functions in the synapses [37, 38].

A growing number of studies indicate that direct and specific NT-lipid interactions could affect the signaling process in addition to the well-documented lipid-protein interactions discussed above. However, due to the sheer amount of evidence that backs up the protein-centric view, too little attention has traditionally been given to the fact that lipids and membrane surfaces also affect NT diffusion or dynamics. Therefore, this perspective aims to widen the protein-centric view of synaptic neurotransmission to include also direct NT-lipid interactions. By examining the published studies on the topic and connecting the dots, it becomes evident that NTs are affected by the membrane environment at every stage of their lifecycles.

## Evidence of Direct Neurotransmitter-Lipid Interactions

**Fig. 1:**
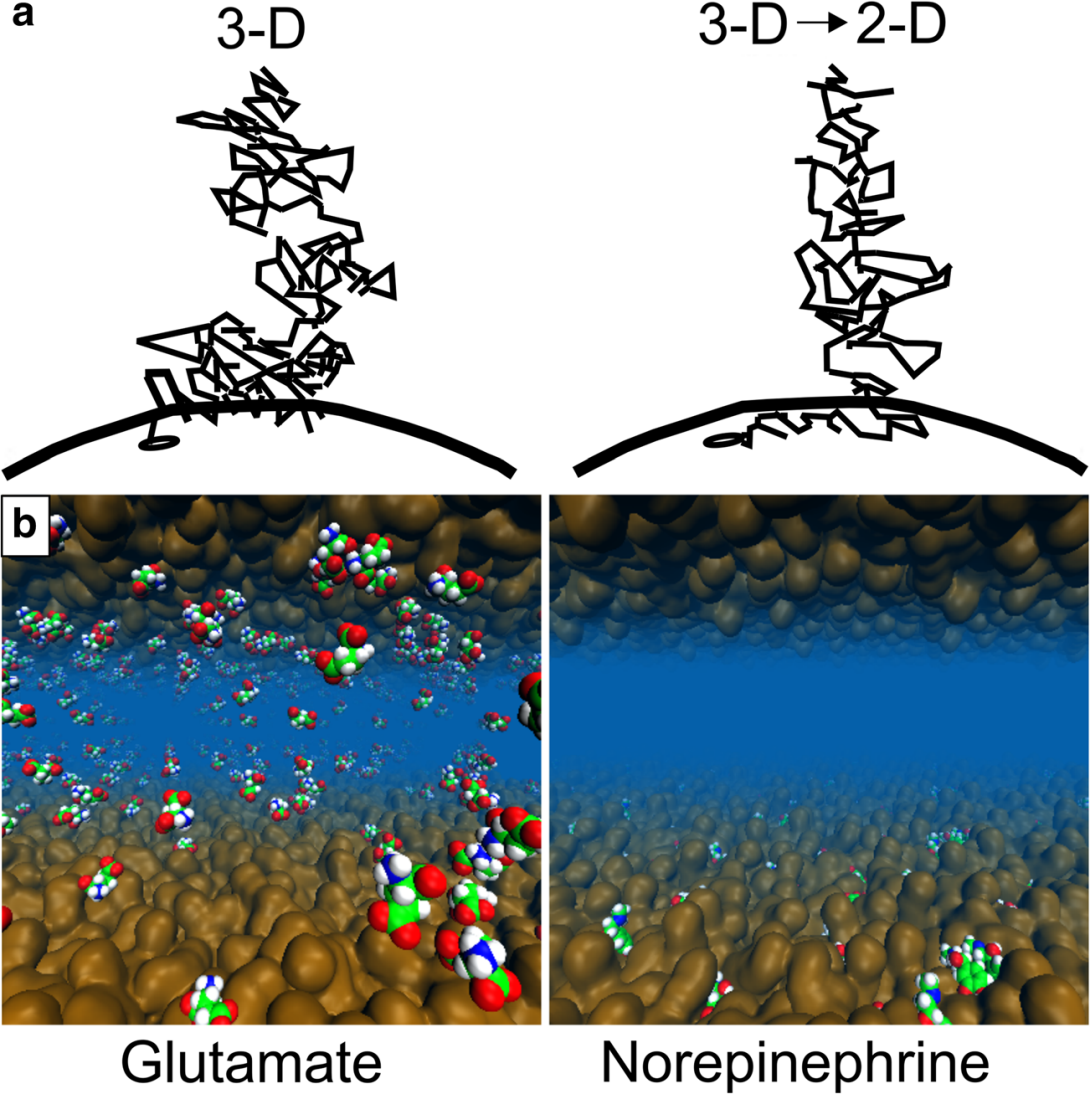
The effect of the membrane on small molecule diffusion and receptor entry. **a** The ligands can either diffuse in 3D towards their membrane-bound receptors (black dot; left) or the 3D diffusion can transform into 2D diffusion along the membrane plane (curved line) prior to the receptor binding (right). Reproduced with the permission from ref. [40]. Copyright 2009 Elsevier. **b** The membrane (brown opaque surface) adherence (lipophilicity) or repulsion (lipophobicity) of neurotransmitters (CPK models) is demonstrated for the norepinephrine (right) and glutamate (left), respectively, using atomistic molecular dynamics simulations. Reproduced with modifications from ref. [42]. Copyright 2016 Postila et al. (https://creativecommons.org/licenses/by/4.0/legalcode)

It is well established that hydrophobic (“water-hating”) small molecules can bind on the cell membrane and, then, diffuse along its plane towards their membrane-embedded receptors (Fig. 1a) [39–41]. Accordingly, it comes to reason that the membrane has another integral and direct function in the synaptic neurotransmission that should not be overlooked; i.e., the NTs are either attracted (lipophilic) to or repelled (lipophobic) by the outer leaflet surface of the postsynaptic cell membrane [42]. However, water-soluble molecules with an amphipathic structure are also known to partition into a lipid bilayer, thus being lipophilic. Amphipathic molecules typically locate to the membrane-water interface (e.g., [43–47]) while hydrophobic molecules locate deeper into the hydrocarbon core of the membrane.

**Fig. 2:**
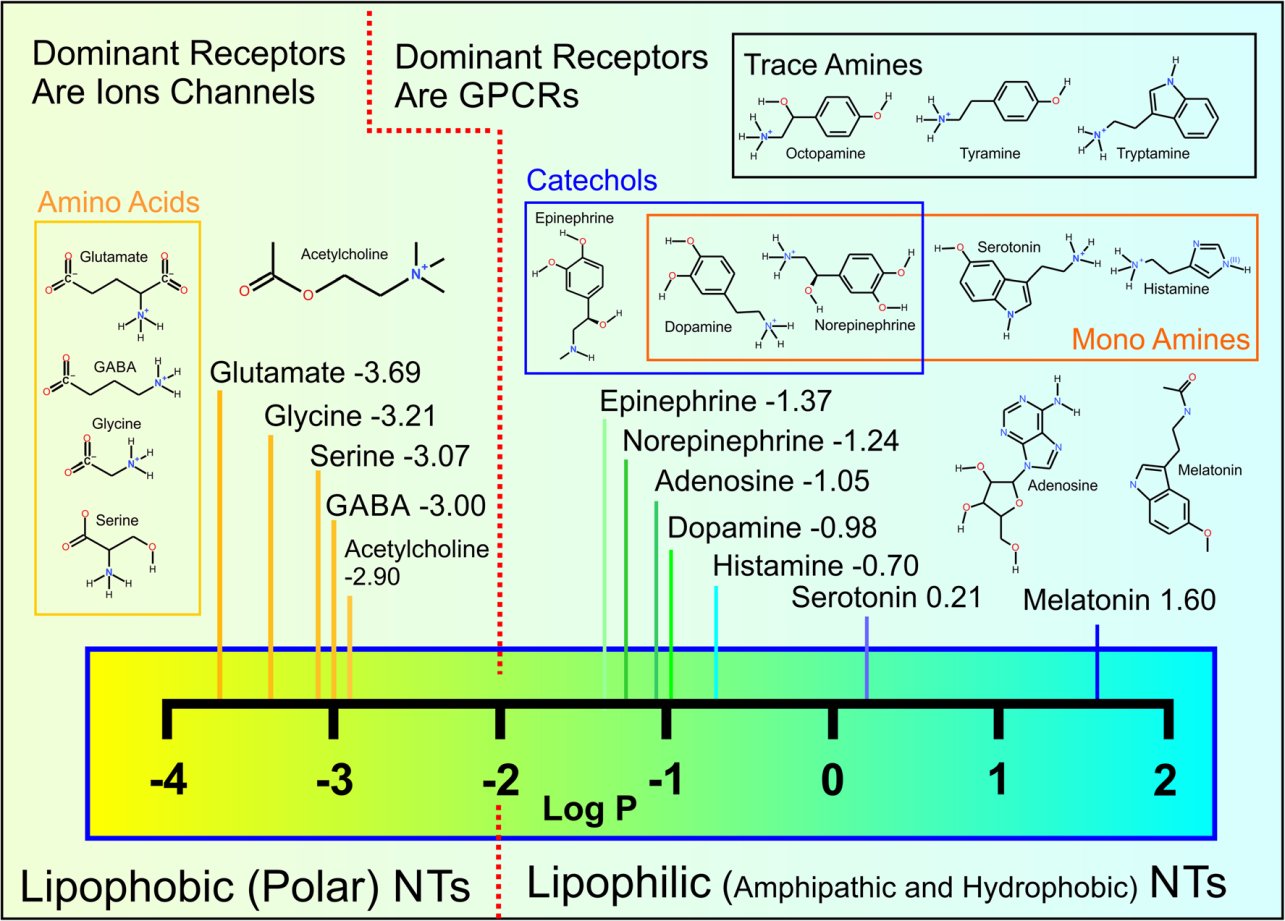
Chemical structures and log *P* values of non-peptidic neurotransmitters. The given log *P* values are experimental except for acetylcholine (https://pubchem.ncbi.nlm.nih.gov/). The log *P* values suggest that the neurotransmitters (NTs) belong to either lipophobic (− 3.69 to − 2.90) or lipophilic (− 1.37 to 1.60) categories. In reality, most of the NTs are amphipathic molecules with both hydrophilic (“water-loving”) or hydrophobic (“water-hating”) groups whose combined effect determines how likely they are to remain in the water phase or to aggregate on the membrane, respectively.

The membrane affinity or lipophilicity of small molecules such as drugs is reflected in their high octanol/water partition coefficients or log *P* values. Non-peptidic or conventional NTs are no exception to this fundamental rule, and their typical log *P* values (Fig. 2) follow the suggested membrane-water phase sorting paradigm (Fig. 1b) [42]. It is, however, an understatement to say that octanol, which is used in the log *P* assay, is too simple a model system for estimating the nuances of NT-membrane adherence that takes place with a complex cell membrane housing diverse sets of lipid species (Fig. 1) [48]. This is underscored by the fact that there exist ~ 21,000 lipid species (The Lipid Maps Structure Database, http://lipidmaps.org/data/structure/), which can be arranged into a myriad of combinations. Thus, not surprisingly, the partitioning of drugs into biological membranes is much higher than predicted by log *P* values [49].

Endocannabinoids such as anandamide, which are derived directly from fatty acids and act as retrograde transmitters [50], deep membrane penetration, and lipid-like dynamics, are to be expected, but the level of membrane permeation or adherence is less evident for the conventional NTs. In fact, several computational and experimental studies have provided solid evidence on the effects of specific NT-lipid interactions and, moreover, highlighted the potential importance of membrane lipid composition (MLC) imbalances for neurological diseases [42, 48, 51–67].

**Fig. 3:**
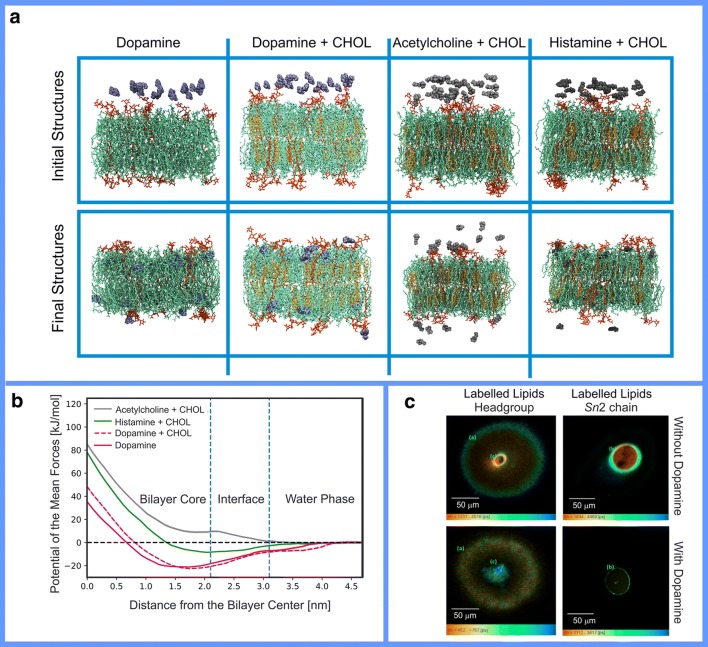
**a** Snapshots of the initial (0 ns) and final configurations (500 ns) obtained in molecular dynamics (MD) simulations of lipid bilayers (stick models) composed of POPC (1-palmitoyl-2-oleoyl-sn-glycero-3-phosphocholin; green), CHOL (cholesterol; orange), ganglioside GM1 (monosialotetrahexosylganglioside; red), in the presence of dopamine, acetylcholine, and histamine (blue or gray CPK models). Water is omitted for clarity. Reproduced with the permission from ref. [57]. Copyright 2018 Elsevier. **b** Free energy profiles of the neurotransmitters translocating thorough the lipid bilayer indicate that dopamine preferred a location at the core of the bilayer below the lipid headgroup region. The center of mass of the bilayer is at 0 nm. Vertical blue dashed lines show approximate regions of the bilayer hydrocarbon core, the membrane-water interface, and water. Reproduced with the permission from ref. [57]. Copyright 2018 Elsevier. **c** Fluorescence lifetime imaging microscopy images of giant lipid vesicles containing nitrobenzoxadiazole (NBD)-labeled lipids at the headgroup or last carbon of the Sn2 chain in the absence and presence of dopamine. The images show dopamine aggregation at the membrane interior. Reproduced with the permission from ref. [64]. Copyright 2017 the American Chemical Association

In this respect, the lipophilic dopamine (Fig. 2) is possibly the most studied small molecule NT. Dopamine was confirmed to partition preferentially onto the membrane based on both atomistic molecular dynamics (MD) simulations (Fig. 3a,b) and experimental monolayer studies (Fig. 3c) [60]. The subsequent simulations with an alternative molecular mechanics force field methodology (OPLS vs. CHARMM) provided similar observations [42, 53]. These theoretical studies indicating strong dopamine-membrane association were further validated by calorimetric [62], nuclear magnetic resonance spectroscopy [63], and fluorescence microscopy [64] experiments.

Similarly, melatonin has been shown to partition onto lipid bilayers via MD simulations, neutron scattering and diffraction, infra-red spectroscopy, fluorescence spectroscopy, calorimetry, and Langmuir-Blodgett monolayer study [51–53, 65–67]. Serotonin was shown to interact with lipids in both theoretical and experimental studies [54, 68]. Adenosine, epinephrine (or adrenaline), and norepinephrine were shown to interact with lipids using MD simulations [42]. Trace amine: tyramine, octopamine, and tryptamine, having similar chemical structures to the above-discussed NTs, were also shown to have an affinity towards different membranes [55]. Finally, short peptidic NTs methionine-enkephalin and leucine-enkephalin were shown to adhere to the membrane surface [61].

Following their low log *P* values (Fig. 2), polar or charged NTs such as γ-aminobutyrate (GABA), glycine, acetylcholine (Fig. 3), and glutamate are not observed to aggregate preferentially at the water-membrane interface in the MD simulations [42]. Despite this, sensitive experimental techniques show that even these lipophobic NTs form both attractive and repulsive interactions with the lipids [56]. Importantly, the membrane partitioning of NTs is modified by the presence of charged lipids or divalent cations [56].

In particular, GABA and glutamate were shown to interact with the lipid bilayers in the presence of Ca^2+^, while acetylcholine in these conditions was repulsed [59]. Next, acetylcholine was shown to be attracted towards bilayers containing negatively charged lipids [42, 56, 59] and repulsed by a bilayer composed of zwitterionic lipids [56]. Similarly, zwitterionic NTs GABA and glycine were attracted towards bilayers containing anionic lipids and were hardly affected by neutral bilayers [56]. Finally, anionic glutamate was repulsed from negatively charged lipids and weakly attracted by a neutral bilayer [56]. It is noteworthy that these interactions lead to increased concentration of polar NTs at the membrane hydration layer [56] without penetration into the membrane core as is observed with the amphipathic NTs [42].

A distinct subset of small non-peptidic NTs are gaseous molecules such as nitric oxide (NO), carbon monoxide (CO), and hydrogen sulfide (H_2_S) [69, 70]. The solubility of these gasses in organic solvents is higher than that of water; thus, their concentration in the lipid phase is relatively high. For example, the concentration of NO in the lipid bilayers is 4.4-fold higher than in water [71], and the concentration difference is 2.2-fold for H_2_S [72]. Free energy calculations based on biased MD simulations are in agreement with these experimental results for both NO [73] and H_2_S [74]. The partitioning of CO into lipid bilayers has been studied using MD simulations, which indicated similar behavior for NO and O_2_ [75]. This is not surprising considering that charge separation in CO is 0.021e and in NO 0.028e. It is also worth mentioning that xenon (Xe), which is a gas with anesthetic properties, is highly soluble in lipid bilayers and most likely acquires its analgesic effects by targeting membrane proteins [41, 76].

## Synaptic Receptor Types vs. Lipid-Neurotransmitter Association

The non-peptidic signal molecules can be divided roughly into two distinct categories based on the experiments: membrane-binding (lipophilic) and membrane-nonbinding (lipophobic) NTs (Fig. 1) [42]. Those NTs such as dopamine with aromatic ring systems, apolar, or otherwise lipophilic/hydrophobic profiles adhere onto the membrane surface (see dopamine in Fig. 3 and norepinephrine in Fig. 1b). Not surprisingly, small lipophobic/hydrophilic NTs such as glutamate (Fig. 1b) or acetylcholine with polar or charged moieties prefer to stay in the water phase instead of adhering onto the membrane (see acetylcholine in Fig. 3). This division of NTs into lipophilic and lipophobic groups is produced effectively by MD simulations with various lipid bilayer models [42], but it can be crudely deduced by simply relying on standard log *P* values as well [42] (Fig. 2).

**Fig. 4:**
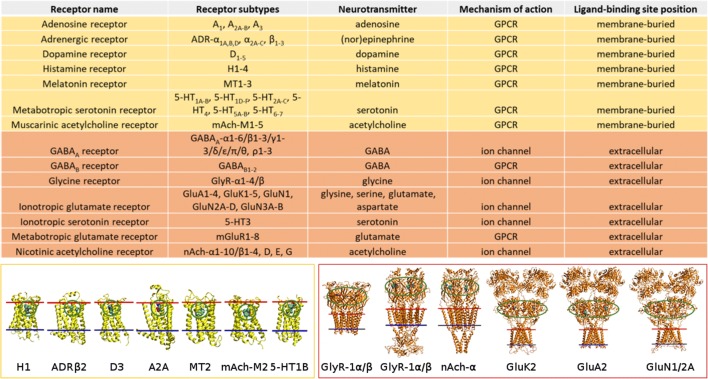
The positions of ligand-binding sites of conventional synaptic receptors in the postsynaptic membrane. The approximate positions of the ligand-binding sites of G protein-coupled receptors (GPCRs) (yellow) and ion channel-forming receptors (orange) are circled green in the protein 3D structures (cartoon models). The inner and outer bilayer leaflets are shown with blue and red lines, respectively. Reproduced with modification from ref. [42]. Copyright 2016 Postila et al. (https://creativecommons.org/licenses/by/4.0/legalcode)

**Fig. 5:**
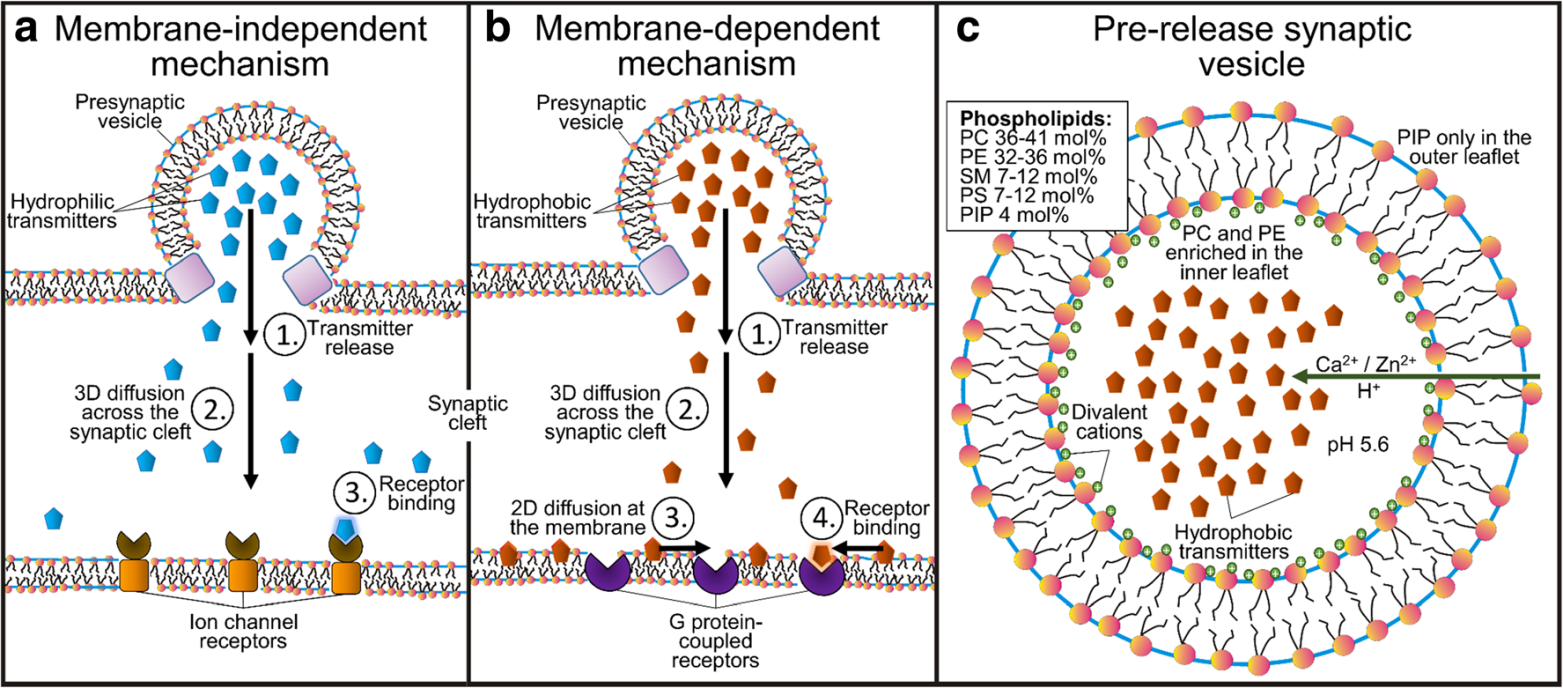
Synaptic neurotransmission models. Left panel—membrane-dependent model: (1) release of lipophilic neurotransmitters (NTs), (2) diffusion across the synaptic cleft, (3) binding onto the postsynaptic membrane surface and 2D diffusion on the membrane plane, and, finally, binding into the receptors. Middle panel—membrane-independent model: (1) release of lipophobic NTs, (2) diffusion across the synaptic cleft, and binding into the receptors. Right panel—the presynaptic vesicle with its known lipid composition [77, 78]. Reproduced with the permission from ref. [58]. Copyright 2017 American Chemical Society

The selective adhesion of NTs at the water-membrane interface is important due to the placement of their receptors’ ligand-binding sites in respect to the synaptic membrane (Fig. 3) [42]. Lipophilic NTs bind preferentially onto the membrane surface from where they can diffuse along the membrane plane towards their membrane-buried binding sites of GPCRs (Figs. 4 and 5). Consequently, the 3D diffusion is transformed into planar 2D diffusion, facilitating presumably faster and more coordinated NT-receptor binding [40]. The most hydrophilic NTs remain preferentially in the water phase without notable membrane surface aggregation [42]—an arrangement that should make the entry into their receptors’ extracellular ligand-binding sites faster and energetically more favorable than if the molecules would adhere on the membrane (Figs. 4 and 5). In effect, the membrane sorting places the released lipophilic and lipophobic NTs closer to their receptors’ binding sites than would otherwise be expected from a completely random 3D diffusion [40].

Based on the lipophilic/lipophobic membrane sorting and established locations of receptors’ ligand-binding sites, the neurotransmission may be divided into (1) membrane-independent and (2) membrane-dependent mechanisms (Fig. 5) [42]. Accordingly, the polar/charged amino acids such as glutamate, glycine, and serine, or the amino acid-like GABA and acetylcholine would not adhere onto the synaptic membrane as they are destined to enter receptors with extracellular ligand-binding sites. Reciprocally, most hydrophobic NTs such as dopamine, its precursor l-dopa, norepinephrine, epinephrine, adenosine, melatonin, and serotonin partition on the membrane, where the GPCRs have membrane-buried ligand-binding sites (Fig. 4).

Various simulation studies [42, 54, 60, 61] and biophysical experiments [51–54, 62–67] corroborate this mechanistic division; however, there are notable exceptions to the rule when focusing on the available receptor protein structures and possible MLCs.

Firstly, serotonin receptors have both extracellular and membrane-buried ligand-binding sites (Fig. 4), although the NT has a notably high log *P* value (Fig. 2) and prefers the membrane surface to the water phase in both simulations and experiments [42, 79]. The membrane preference of serotonin could be overcome by only increasing its secreted levels in those synapses where membrane adherence is unfavorably affecting the receptor entry. Secondly, although acetylcholine binds into the extracellular ligand-binding sites of both nicotinic acetylcholine receptor (Fig. 4) and acetylcholinesterase, the positively charged NT also has to enter the membrane-buried binding site of muscarinic acetylcholine receptor [80] (Fig. 4). The lack of membrane adherence seems unlikely to be overcome by only increasing the acetylcholine levels; however, acetylcholine could be forced to bind onto a membrane containing anionic lipids. In other words, the inconsistency of acetylcholine binding sites could, in theory, be explained by MLC differences between those synapses housing either nicotinic or muscarinic acetylcholine receptors (Fig. 4). By regulating the MLC tightly, the NT entry would follow either the membrane-independent or membrane-dependent mechanism [42] (Fig. 4).

## The Effect of Membrane Lipid Composition on Neurotransmitter Dynamics

The adjacent neurons form a synapse specialize in the release of specific NTs; for example, the neuromuscular junctions are packed with nicotinic acetylcholine receptors (Fig. 4) that selectively bind acetylcholine [81]. This means that the protein machinery both at the pre- and postsynaptic membranes is tightly regulated to match the needs of the secreted NT types in each synapse. Likewise, it comes to reason that also the MLCs would be specific for these different synapses and optimized to assure fast and efficient signaling with the NTs in question.

Recent lipidomics studies have led to the vast and rapid expansion of the available data on various organs, tissues, cell types, and cell organelles [82, 83]. Similarly, data concerning lipidome changes in pathological states, including neurological and mental disorders, provide new insight into the possible role of lipids in neuronal tissues [84–89]. Although no direct link has been established so far, it is easy to fathom how an imbalance in the strength of NT-membrane partitioning could contribute to, for example, major depressive disorder or Parkinson’s disease. Typically, both conditions are treated by increasing the concentration of the responsible NTs in the neurons. With depression, antidepressants increase either the level of serotonin or dopamine at the synapse by blocking their transport or by inhibiting their catabolism. With Parkinson’s disease, the dopamine precursor l-dopa, which, unlike dopamine diffuses across the blood-brain barrier, is administered to increase the effective dopamine levels. In theory, the local concentration of the NTs either at the membrane surface or in the water phase could be negatively affected by too strong or weak membrane association; thus, the MLC would be at least a partial culprit for the shortage of NTs that can bind into the receptors.

**Fig. 6:**
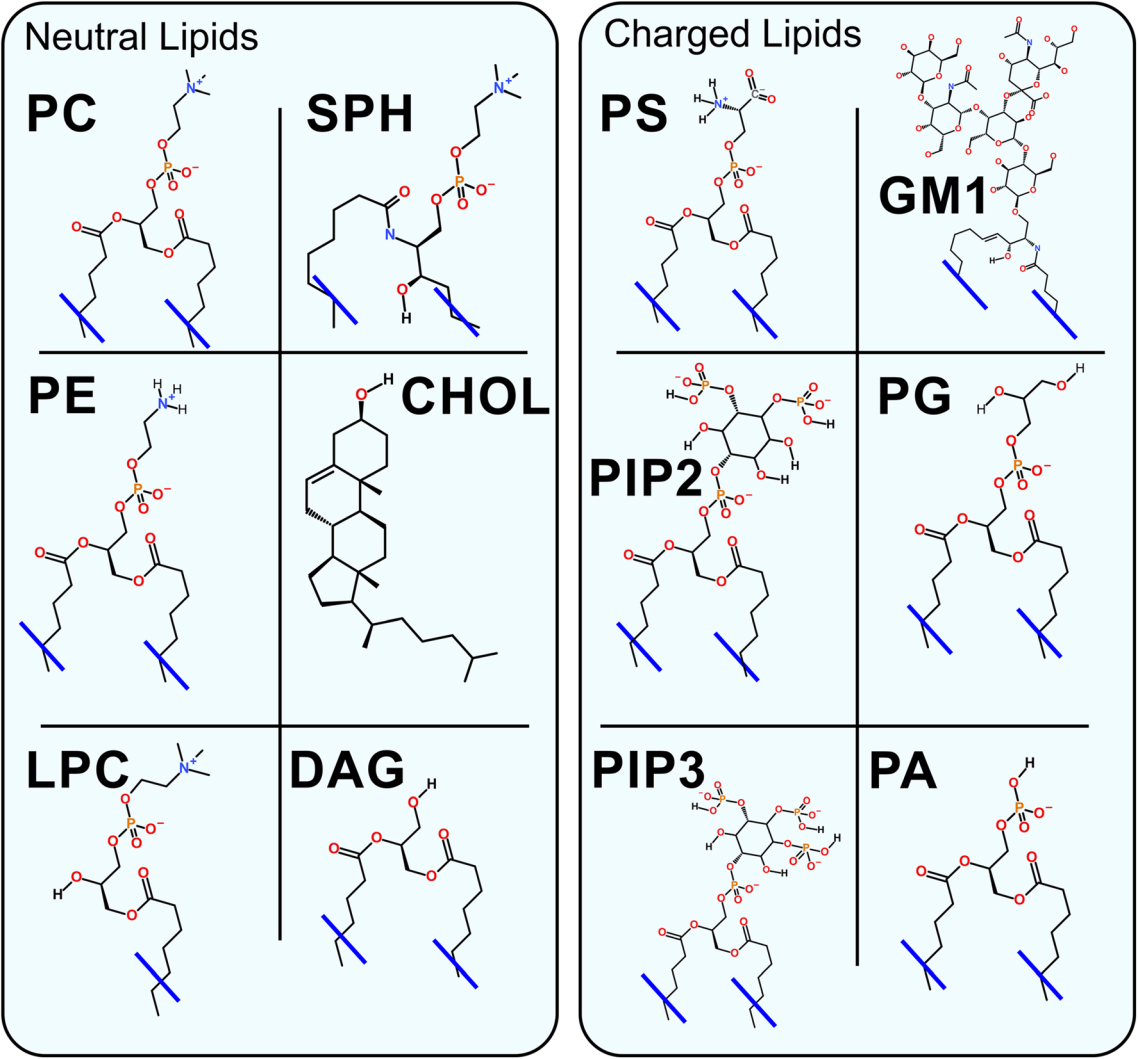
Chemical structures of the most common lipids. PC, phosphatidylcholine; PE, phosphatidylethanolamine; SPH, sphingomyelin; CHOL, cholesterol; LPC, lysoPC; DAG, diacylglycerol; PS, phosphatidylserine; GM1, monosialotetrahexosylganglioside; PG, phosphatidylglycerol; PIP2, phosphatidylinositol bisphosphate; PIP3, phosphatidylinositol triphosphate; PA, phosphatidic acid

Despite the rapid progress of lipidomics analysis [90–93], the MLCs for specific synapses or neurons are not particularly well characterized to date. The synaptic membrane leaflets are expected to remind a typical animal cell: the extracellular leaflet being neutral and composed mainly of sphingolipids (SPH; Fig. 6), saturated phosphatidylcholine (PC; Fig. 6), and CHOL (Fig. 6); and its more anionic counterpart the intracellular leaflet containing more negatively charged lipids such as phosphatidylserine (PS; Fig. 6), PIP (Fig. 6), and phosphatidylglycerol (PG; Fig. 6) [94]. The only notable charged lipid species of the extracellular leaflet, whose levels are moderately high in the neurons where their molar fraction is in the order of few mol%, are the glycolipids including gangliosides and sulfogalactosyl ceramides [95]. As their concentration can reach even 30 mol% [96, 97], glycolipids such as monosialotetrahexosylganglioside (GM1; Fig. 6) are potentially a vital membrane component affecting the NT dynamics via charge-related effects. Moreover, an aberration in sulfogalactosyl ceramide content in neurons was observed for the case of few neurodegenerative disorders [98, 99].

In fact, studies have shown that the main lipid classes in the synaptosome—a structure containing both the pre- and postsynaptic membranes, synaptic vesicles, and possibly fragments of other cells—follow this general pattern of animal cell MLC. Nevertheless, notable differences between the synaptosome and whole brain lipids have been observed [100]. A more accurate lipidomics study of the synaptosome, in which ~ 80 lipid species were identified, showed significant differences in the MLC of the synaptosome in comparison with the synaptic vesicle [101] (Fig. 4). Another recent lipidomics study of the postsynaptic membrane showed large changes in the lipid profile during brain development. It was shown that the amount of CHOL, SPH, and ether lipids increases with time. Moreover, the decrease of the number in short tails and an increase in the number of tails with six double bonds were observed [102]. In the central nervous system, CHOL originates from in situ performed synthesis [10], which takes place predominately in astrocytes [103]. Thus, the decrease of CHOL synthesis by astrocytes could lead to the impairment of the brain development and decrease of neuron growth in co-culture of neurons with astrocytes [104].

The atomistic MD simulations [42, 54, 60, 61] (Fig. 3a, b) and experiments [51–56, 62–68] alike show that the lipid assortment indeed affects NT dynamics profoundly. Majority of the non-peptidic NTs are attracted towards the anionic membrane models to a varying degree [42, 56, 59, 62]. That includes even the most charged and hydrophilic NTs such as GABA or glycine as they show a moderate level of adherence on the membrane models containing anionic lipid PS (Fig. 6), PG (Fig. 6), and phosphatidic acid [56]. This adherence onto the anionic membranes in comparison with the neutral membrane models is explained by the prevalent charge factor and electrostatic interactions: the anionic lipids attract the positively charged, amphipathic or zwitterionic NTs even if this charge-based attraction does not necessarily assure complete membrane preference over the water phase or full lipophilicity [56]. The only exception seems to be the anionic glutamate, which is repulsed by the anionic lipids [56].

With dopamine and histamine, the presence of physiologically relevant glycolipid GM1 (Fig. 6) lipid species was enough to enhance their already notably strong membrane preference based on the MD simulations [57]. The same effect could not be replicated with the acetylcholine, whose binding to muscarinic receptors (Fig. 4) should benefit from the membrane partitioning as suggested by the dualistic neurotransmission model (Fig. 5) [42]. The membrane adherence of acetylcholine is achieved with a membrane model containing PS in the simulations [42] and PS or PG experimentally [56]; however, these lipid types are more endogenous to the intracellular leaflet of the cell membrane than the typical extracellular leaflet. Thus, if stable membrane adherence of acetylcholine is indeed a prerequisite for the entry into the membrane-buried ligand-binding site, the membrane housing the muscarinic receptors should contain lipids that are more anionic than the GM1 (Fig. 6).

It is well established that CHOL (Fig. 6) decreases the transport of gasses through lipid bilayers [41, 105–107]. NO diffusion in the lipid bilayers can be slowed down by 20–40% depending on the MLC [108, 109] and also the permeability coefficients of NO can be reduced by 17% [110]. MD simulations and free energy calculations indicate that CO and NO behave similarly in the CHOL-rich membrane environment [111]. Nevertheless, the diffusion of NO through biological systems is fast (2.2 × 10^−5^ cm^2^/s) [112–114] and, moreover, the process is likely facilitated by aquaporins [73]. Thus, gaseous NTs should be able to diffuse and bind into their specific receptors regardless of their positioning in respect to the lipid bilayer.

## Neurotransmitter Transporters: Lipid-Assisted Neurotransmitter Entry?

Synaptic receptors (Fig. 4) are not the only proteins in the synapse that bind and interact with the NTs. The neurotransmitter transporters with membrane-buried ligand-binding sites also bind NTs in order to transport them away from the synaptic cleft after their release and elicited function [115]. For example, with glutamate, the transport must be expedient due to its potentially toxic effects of the prolonged presence in the synapse [116]. The role of the transporters is complex because the NTs can also move in reverse through them in certain conditions [117] instead of relying on the presynaptic vesicle release (Fig. 5). Until very recently [118, 119], mechanistic insight into the neurotransmitter transporters has relied on X-ray crystallographic structures of their bacterial counterparts such as leucine transporter [115].

The paradox is that even the most hydrophilic or lipophobic NTs (Fig. 2) that are not prone to adhere on the membrane (Fig. 3) must be transported actively away from the synapse through the transporters with the membrane-buried binding sites. The increased anionic or hydrogen-bonding capable lipid content within the membrane patches surrounding the transporter proteins, in theory, could assist the entry into the buried binding sites. While there is no evidence on the implied effect of direct NT-lipid interaction on the transport, the MLC has been shown to influence bacterial aspartate transporter conformation and function via specific pi-cation interactions [120]. Furthermore, the presence of charged amino acid residues at the water-membrane interface could assist at the early stage of NT entry into the transporters.

Acetylcholine, whose efficient entry into the muscarinic receptors (Fig. 4) seems to require highly anionic MLC based on the MD simulations [42], remains an odd example also when inspecting its removal from the synaptic cleft. Acetylcholine is catalyzed into choline and acetate by the acetylcholinesterase and, notably, the enzyme’s ligand-binding site is extracellular and, thus, the positively charged NT removal is not directly dependent on its membrane adhesion.

## Presynaptic Vesicle: Three Fail-Safes for Assuring Efficient Neurotransmitter Release

The pre- and postsynaptic membranes are not the only lipid bilayer surfaces (Fig. 4) that NTs interact with during the neurotransmission. Upon the arrival of the action potential, the aggregation of PIPs (Fig. 6) instigates the protein assembly leading to the release of the NTs from the presynaptic vesicles into the synaptic cleft [13]. For this release to function fast and efficiently, the NTs, no matter how lipophilic (Fig. 2), should not aggregate excessively on the inner leaflet of the presynaptic vesicle as this would hinder the signaling overall.

There are at least three fail-safe mechanisms for preventing the unwanted membrane aggregation onto the inner leaflet of the vesicle (Fig. 5) [58]. Firstly, the lumen of the vesicle is kept relatively acidic (pH 5.6) in comparison with the physiological pH (7.4). The low pH assures that the negatively ionizable parts of lipid headgroups and NTs are more likely to be fully protonated/neutral and, thus, remain not attracted to each other [121]. The generated proton gradient also assists in the transport of the NTs into the vesicles by vesicular neurotransmitter transporters [121]. Secondly, the MLC of the vesicle’s inner leaflet is composed of mainly neutral lipid species such as PC and phosphatidylethanolamine (PE) (Fig. 6), whereas the highly anionic PIP lipid is present in the outer leaflet [77, 78]. Thirdly, positive and divalent cations Ca^2+^ and Zn^2+^ are actively pumped inside the vesicle where they can adhere on the anionic headgroups of the membrane lipids and neutralize the water-membrane interface [121, 122].

The charged MLC is crucial for the membrane adherence of NTs from both ends of the hydrophilicity and hydrophobicity scale [42, 51–56, 60–67]. Charge neutrality of the headgroup region of the membrane assists the release of all non-peptidic NTs regardless of their propensity to partition on the membrane in general (Figs. 3 and 6). This is more urgent when dealing with very lipophilic NTs because they tend to aggregate onto the water-membrane interface regardless of the MLC [42, 53, 60, 61, 65, 66] and, for example, dopamine almost entirely aggregates on to the membrane in the MD simulations [42, 60] (Fig. 3). This membrane preference of dopamine is predicted to be prevented almost entirely by the neutral MLC, and high calcium levels present inside the presynaptic vesicles based on the simulations and free energy calculations [58]. Nevertheless, the mechanisms for detachment of lipophilic NTs and drugs from various membranes in the synapse remain mostly unknown and, thus, require more scrutiny in the future.

## Role of Membranes in Intracellular Neurotransmitter Metabolism

What is also not well recognized in the current literature is that both the synthesis and degradation of amphipathic NTs happen within the context of membranes. While there might not exist direct evidence of membrane assistance in these intracellular metabolic processes, for example, in the case of dopamine, the essential proteins are membrane-bound or membrane-associated based on the latest research.

Dopamine, the first catechol NT at the biosynthetic pathway, is synthesized from the amino acid tyrosine in two steps: (1) the tyrosine hydroxylase converts it to l-dopa, and (2) the l-dopa decarboxylase converts it into dopamine. Although both enzymes were previously considered to be cytosolic, recent studies have indicated their association with membranes: the tyrosine hydroxylase binds to liposomes [123], and the l-dopa decarboxylase associates generally with the membranes in the cellular environment [124]. In the next step, dopamine is used as a substrate in the synthesis of two other catechol NTs: (1) norepinephrine and (2) epinephrine. The first step of the conversion is performed by the dopamine-β-hydroxylase enzyme that has both water-soluble and membrane-bound forms [125]. An alternative pathway of dopamine biosynthesis utilizes the cytochrome P450 enzyme [126], which is an established integral membrane protein. Degradation of dopamine after its reuptake is an essential process due to the toxicity of its oxidized form [127]. The initial steps of dopamine degradation are suggested to be performed by four different enzymes, including the membrane-associated monoamine oxidize [128] and uridine diphospho-glucuronosyltransferases [129], and, finally, catechol-o-methyl transferase (COMT) [130] and sulfotransferase [131] that have both membrane-bound and water-soluble forms.

## The Effect of Membrane Sorting for Drugs and Beyond

The studies reviewed in this perspective indicate that the NT-membrane interactions are likely to be tightly regulated and, therefore, a crucial part of the synaptic neurotransmission. This level of organization and coordination is needed because the NT diffusion across the synaptic cleft (20–30 nm) takes only a few microseconds [132]. Moreover, the membrane-based sorting (Fig. 4) not only affects neurotransmission efficiency but likely extend to all phases of the NT life cycle, including their release from the synaptic vesicles, diffusion across the cleft, receptor entry or binding, removal from the synaptic cleft, as well as their production and eventual degradation.

The potential role of the membrane sorting (Fig. 5) should not be overlooked with any small molecules or ligands due to the ubiquitous presence of membranes in the synaptic cleft (Fig. 1) or, ultimately, inside or outside of any cell. If a drug is due to enter a cell membrane-buried or an extracellular ligand-binding site (Fig. 5), its binding rate should benefit from the sorting (Fig. 1a) [40, 42]. This is analogous to matching the charge/shape properties between the receptors’ ligand-binding sites with those of the ligands during docking [133–136], docking rescoring [137–139], or drug discovery [136, 140–142]. In fact, there is overwhelming evidence of the affinity of small molecules towards lipids [28], and the MLC is also known to affect drug membrane permeability [143]. The log *P* values, membrane permeation, and lipophilic efficiency are already routinely considered in the medicinal chemistry [144]. The difference between the drugs and NTs is that the synaptic MLCs are fine-tuned through evolution for the unobstructed dynamics of the latter group and not necessarily for the former [42]. However, if a drug binds into the same ligand-binding site as the endogenous ligand (not an allosteric modulator), it is likely to have a similar charge profile for interacting with the membrane. For example, the ligand-binding sites of ionotropic glutamate receptors are highly charged to match the opposite charge profile of its ligands, mainly glutamate [145]—a feature that also affects their dynamics with the membrane (Fig. 1a) [42].

**Fig. 7:**
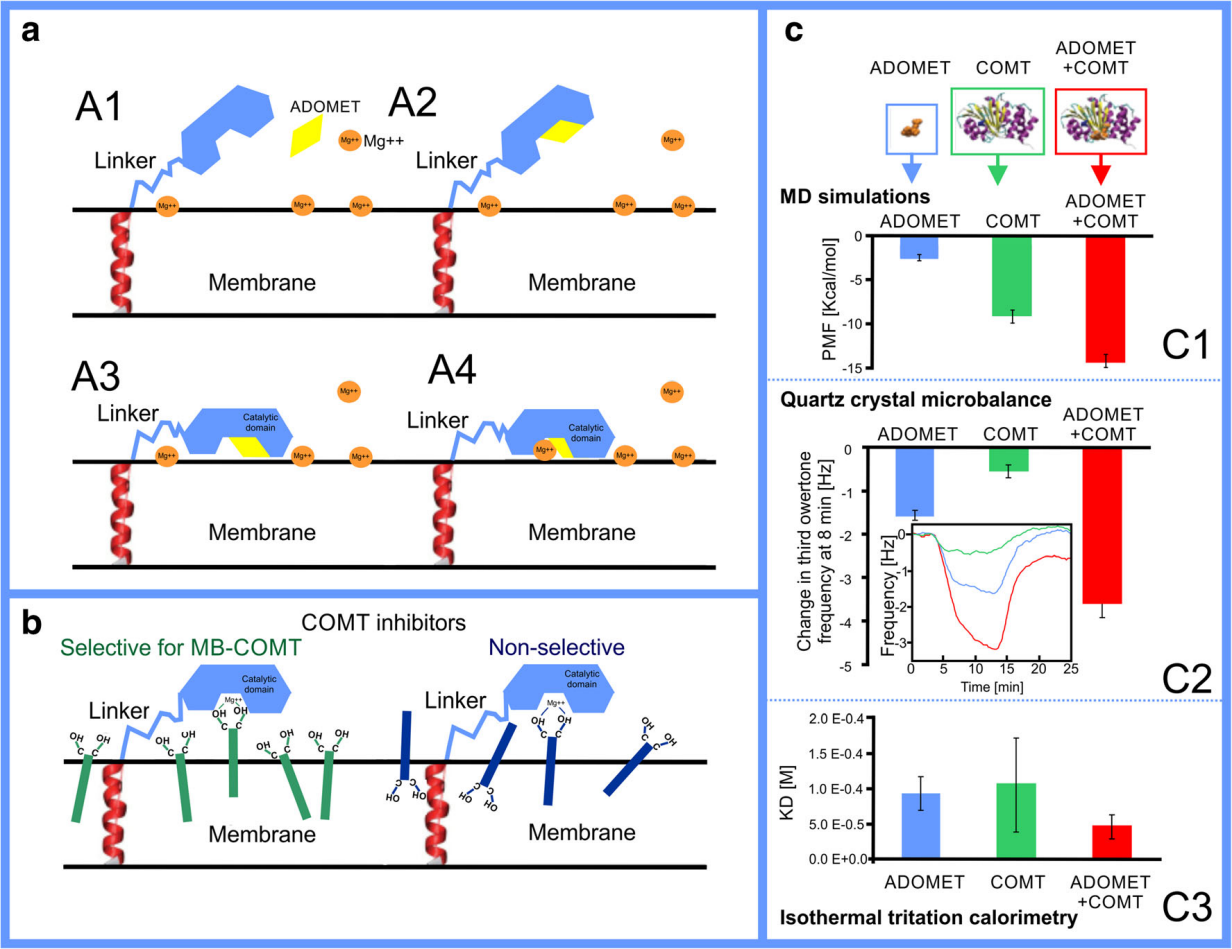
The pivotal role of the membrane in membrane-bound catechol-o-methyl transferase catalysis and selective inhibition. **a** Steps of catalytic mechanism of the membrane-bound catechol-o-methyl transferase (MB-COMT): (A1) the catalytic domain interacts weakly with membrane in the *apo* form; (A2) the cofactor S-adenosyl-l-methionine (ADOMET) binds to the catalytic site of MB-COMT; (A3) MB-COMT in complex with ADOMET opens the catalytic site towards the membrane, which, in turn, allows the protein to bind to the membrane surface; (A4), finally, the MB-COMT binds an Mg^2+^ ion that is already present at the membrane surface. **b** The behavior of MB-COMT selective vs. non-selective inhibitors in the membrane: selective inhibitors orient catechol group towards the water phase and, in contrast, non-selective inhibitors could be oriented less optimally in relation to the MB-COMT catalytic site. **c** The estimations of interactions of the ADOMET and catalytic domain of COMT in complex and separately with lipids indicate that the catalytic domain is preferably membrane-oriented: (C1) the free energy changes when protein is pulled away from the lipid bilayer; (C2) quartz crystal microbalance (QCM) frequency changes during interaction with the lipid bilayer; (C3) dissociation constant (inverse of affinity) from lipid bilayer (vesicle) determined by isothermal calorimetry. Reproduced with permission from ref. [146]. Copyright 2018 the Royal Society of Chemistry

The importance of membranes for drug development was clearly demonstrated in a recent study showing dependency between inhibitor membrane location and orientation in respect to the membrane surface (Fig. 7), and its selectivity towards the membrane-bound form of catechol-o-methyl transferase (MB-COMT) compared with its water-soluble form (S-COMT) [146]. The COMT inhibitors are used together with l-dopa in the treatment of Parkinson’s disease to prevent dopamine deficit [147, 148]. l-Dopa is converted into dopamine inside the neurons, and the role of COMT inhibitors in the drug formulation is to prevent l-dopa degradation. Interestingly, in the brain tissues, the MB-COMT dominates, while in the remaining part of the body, the S-COMT is dominant. For this reason, the development of MB-COMT-specific drugs (Fig. 7b) is beneficial due to the likely reduction in the undesired side effects.

## Conclusions

All four components, the neurotransmitter (NT), the membrane lipid composition (MLC), the shape/charge of the receptor’s binding site, and its location in relation to the lipid bilayer, should match; otherwise, the efficiency of the neurotransmission is bound to suffer. If an NT is due to binding into an extracellular ligand-binding site, it does not adhere firmly on the membrane surface. In contrast, an NT that binds into a membrane-embedded ligand-binding site has a strong tendency to adhere to the membrane as well. This division of neurotransmission into the membrane-independent and membrane-dependent mechanisms is supported by molecular dynamics simulations, X-ray crystallography, log *P* values, and other sensitive biophysical experiments. Even prior to an NT release into the synaptic cleft, prohibitively strong membrane adherence of NTs is prevented by controlling the MLC of the inner leaflet, pH, and ionic content of the presynaptic vesicles. In fact, an NT dynamics is likely to be affected by the membrane environment during their transport out of the cleft as well as their intracellular synthesis and degradation. Collectively, the data supports the view that individual synapses, which are specific for certain NTs, have carefully curated global and/or local MLCs to assure fast neurotransmission and avoid potential disease states caused by NT-membrane mismatches. In short, the membrane should not be seen as a passive barrier or a mere scaffold for proteins, but rather as an active participant or even a nexus that facilitates the fast-paced synaptic neurotransmission.
